# Supplementary material for: Are carbon emissions trading and green financial instruments synergistic? -Comprehensive quantitative research based on content analysis
Source: PLoS One. 2024 Mar 7;19(3):e0298601. doi: 10.1371/journal.pone.0298601 (PMC10919601; doi:10.1371/journal.pone.0298601)
Supplement: S1 Data — (ZIP) [file pone.0298601.s001.zip › Data/地级市Z_F工作报告.zip!/╣π╢1⁄2/╔ε█┌╩╨/└┤╫╘╣1⁄2╓┌║┼í╛┬φ┐╦╩2╛▌═°í┐_2009.docx]

各位代表：

　　现在，我代表深圳市人民政府向大会报告工作，请予审议，并请各位政协委员和其他列席人员提出意见。

　　一、2008年工作回顾

　　2008年是极不寻常、极不平凡的一年。全市人民在党中央、国务院、广东省委、省政府和市委的正确领导下，坚持以邓小平理论和“三个代表”重要思想为指导，以科学发展观统领经济社会发展全局，以解放思想增强发展动力，努力克服国际金融危机和国内特大自然灾害的不利影响，加快经济发展，促进社会和谐，圆满完成了市四届人大五次会议确定的各项目标任务。

　　（一）科学发展水平稳步提高。全年实现本市生产总值7806.5亿元，增长12.1%。人均GDP 达到13153美元，提前两年完成“十一五”规划目标，继续居内地大中城市首位。规模以上工业增加值3527.8亿元，增长12.5%。全社会固定资产投资1467.6亿元，增长9.1%。社会消费品零售总额2251.8亿元，增长17.6%。进出口总额2999.7亿美元，增长4.3%。其中，出口总额1797.4亿美元，增长6.6%，实现十六连冠。全口径财政收入2830亿元，其中属中央收入2029亿元，地方财政一般预算收入800.4亿元，增长21.6%。居民消费价格上涨5.9%，高出预期目标1.9个点，但12月价格涨幅已回落至2.5%。2008年，全市每平方公里GDP产出达到4亿元，比上年提高0.52亿元。万元GDP能耗继续下降，万元GDP水耗下降10.5%；二氧化硫和化学需氧量排放量分别下降9.3%和6.8%。

　　（二）经济调控能力显著增强。在全国较早准确预见到我市面临比1997年亚洲金融危机更严峻的挑战，全面加强了对经济工作的领导，政府主要领导主持了59次经济工作调研、调度和会议，力度之大、频度之密、规格之高，在深圳经济发展史上是罕见的。开展了“工业贸易百人调研服务”活动，及时出台一系列扶持企业和产业发展的政策措施。财政年度投入20.7亿元，设立创投引导资金和企业互保金贷款代偿资金，建立再担保体系，降低中小企业上市融资成本。在全国首发中小企业短期融资券，推动中小企业集合发债。加快城市空间二次开发，完善产业用地出让政策，将工业和产业用地出让时间大幅压缩40天。解决了19家上市公司的用地问题，启动创新产业用房建设，扩大产业发展空间。政府垫付20亿元电价调节金，确保电力供应，降低用电成本。将企业堤围防护费最高征收标准降低80%，财政出资2.2亿元支持外贸出口企业开拓国际市场，降低企业部分社会保险费缴交标准一年，有效减轻企业负担近20亿元以上。对拥有自主知识产权的品牌企业和骨干企业实行“服务直通车”，对重点企业配备由政府领导担任组长的服务小组，着力解决企业创新发展中遇到的各种问题。认真落实国家房地产有关政策，依法引导房地产市场健康发展。这些措施有效改善了企业的生产经营环境，在极其困难的条件下成功遏制了经济下滑，推动经济运行逐步好转，成为全国抗击金融危机的一个亮点，得到了温家宝总理等中央领导的高度评价。

　　（三）综合竞争力全面提升。经国家发改委批复，我市成为首个国家创新型城市建设试点城市。推出了加快建设国家创新型城市的意见和总体规划，出台33条鼓励政策，优化创新环境，推动高新技术产业新一轮大发展。制定实施了加强高层次专业人才队伍建设的系列政策，成功举办了2008中国国际人才交流大会，组团赴海外大规模招聘人才。中科院深圳先进技术研究院、华大基因研究院等科研机构加快发展，创新基础进一步夯实。全社会研发投入占GDP比重达到3.3%左右，获得8项国家科学技术进步奖，其中一等奖一项。全市发明专利授权量达5404件，增长1.39倍；PCT国际专利申请2709件，占全国44.5%，连续五年稳居全国首位。华为技术有限公司名列全球专利申请排名榜首位。

　　产业结构进一步优化。三次产业结构为0.1∶48.9∶51.0，第三产业比重近6年来首次超过第二产业。高新技术产业成为深圳抵御世界性经济危机的重要力量，全年实现高新技术产品产值8711亿元，增长14.6%，其中拥有自主知识产权的产品占59.1%。金融业稳步发展，年末金融机构本外币存贷款余额分别为1.43万亿元和1.12万亿元，分别比年初增长12%和11%。物流业继续发展，深圳港集装箱吞吐量2141.6万标准箱，连续6年位居全球第四；深圳机场旅客吞吐量2140万人次，连续7年位居内地第四。前海湾保税港区获国务院批准设立。文化产业园区建设稳步推进，一批文化科技型企业加快发展。旅游、服务外包等高端服务业保持较快增长，成功举办第十届“高交会”、第四届“文博会”和首届深圳文化产权交易会等一批品牌展会。

　　产业发展后劲进一步增强。制定出台加快建设现代产业体系暨推进双转移工作的系列政策和支持总部经济发展的措施，加快构建现代产业体系。以高端制造业和高端服务业为重点，实施重大产业项目招商引资计划和招商引资责任制。中芯国际集成电路芯片、杜邦薄膜太阳能电池、大族激光全球制造基地等一批重大产业项目开工建设，航天集团南方总部、赛诺菲巴斯德流感疫苗等项目正式奠基，世纪晶源芯片、日东电工偏光片、深超光电第5代TFT-LCD等项目正式投产，比亚迪双模电动车实现量产，弥补和完善了产业链的关键缺失环节。高新区、光明高新园区、大工业区、软件产业基地、生态精细化工园等重大产业基地规划建设发展顺利，传统产业集聚基地建设步伐加快。

　　（四）改革开放有序推进。积极配合和参与《珠江三角洲地区改革发展规划纲要》的调研起草工作，通过《纲要》正式明确了我市作为国家综合配套改革试验区、全国经济中心城市、国家创新型城市、中国特色社会主义示范市和国际化城市的新使命、新定位，为我市深化改革开放、推动科学发展赢得了新契机。我们正在认真制定和完善我市综合配套改革总体方案，力争在一些重点领域和关键环节先行先试，取得新突破。认真开展解放思想学习讨论活动，成立了市委市政府改革领导小组，调整了改革部门，以世界先进城市为标杆，创新思路、推进改革、扩大开放。市属国有企业三项制度改革阶段性任务基本完成，国有独资公司董事会建设试点工作取得成效，国有资产监管体系不断完善。行政审批制度改革继续深化，积极探索相对集中行政许可，加强并联审批和协同办理，完成全部非行政许可审批和登记事项的清理工作。以全新体制建立的光明新区实现正常运作和健康发展。积极探索公务员分类管理改革，聘任制公务员试点工作稳步开展，公安系统公务员专业化改革深入推进。事业单位改革继续深化，法定机构试点工作、事业单位法人治理结构改革等取得阶段性成果。积极推动和规范社会组织发展，创新社会组织登记管理体制，被列为全国全省社会组织“改革创新综合观察点”。创新人口管理模式，全面推行居住证制度，年内居住证办证量突破700万张。

　　开放水平不断提升。中国—越南（深圳—海防）经贸合作区项目正式启动，与国家发改委、韩国SK电讯株式会社签署了加强信息技术创新合作谅解备忘录。积极落实国家鼓励出口的各项政策，研究出台支持外贸企业发展的具体措施，加强各类贸易摩擦应对工作，组织实施一系列境内外经贸推介活动，积极帮助企业拓展市场。深港、深澳合作会议首次在深圳举行，开启了深港、深澳合作新的一页。“深港创新圈”加快发展，深港河套地区综合开发研究取得新突破，两地联手应对金融危机取得共识，深港更紧密合作关系进一步巩固。对口支援和区域合作向纵深推进，深汕鹅埠产业转移园进入实质合作阶段，省外各深圳工业园建设加快推进，对内地的辐射带动能力进一步增强。圆满完成抗击雨雪冰冻、暴雨洪灾和千里驰援汶川地震灾区等重大抗灾救灾任务，率先提前建成援助四川灾区的活动板房，强力推进甘肃陇南甘南地震灾区恢复重建工作，受到中央领导的充分肯定和灾区人民的高度赞扬，被誉为全国对口支援工作的一面旗帜。

　　（五）城市环境更加完善。城市规划建设管理不断加强。光明新城建设全面启动，四大新城建设取得实质性进展，特区外基础设施和公共设施持续改善，特区内外一体化进程不断加快。城市总体规划和土地利用总体规划完成上报程序，法定图则覆盖面继续扩大。积极创新土地管理模式，加大城市更新改造力度，福田岗厦城中村、南山永新工业区等改造试点项目稳步推进，违法用地和违法建筑受到控制，城中村和社区环境综合整治取得成效，海滨15公里休闲带正式开工建设，道路修缮和二线关环境综合整治基本完成，城市总体形象进一步改善。完善综合执法工作，初步调整了综合执法范围，街道综合执法工作得到进一步加强。公交特许经营改革取得阶段性成果，规划建设三层次公交体系，开通特区外首批5条快速公交线路。特区内500米公交站点基本实现全覆盖，特区外覆盖率达到70%。落实公交燃油价格补贴、公益性补贴13亿元，进一步减轻了市民出行负担。严厉打击非法营运、套牌车等违法行为，出租车管理得到加强。

　　适应经济形势变化，着力加大固定资产投资协调推进力度，全年基本建设投资达到827.8亿元，增长15.9%，创历史新高。广深港客运专线、厦深铁路建设进展顺利，穗莞深城际轨道干线正式开工，城市轨道交通建设按计划稳步推进，地铁设备国产化水平领先全国。深盐二通道主线、盐坝高速C段、南光高速、华为立交一期等建成通车，南坪快速路二期、西部疏港路、丹平路一期等建设加快。盐田港集装箱码头扩建、大铲湾港一期、蛇口港三期工程和国际客运候机楼等项目建成投入使用，深圳机场扩建陆域基本形成。

　　资源能源保障体系不断完善。水库扩容改造全面推进，铁岗水库主体工程基本完工，公明、清林径、东江下矶角等供水工程稳步推进，北线引水、东江水源二期工程加快建设。电网建设投入力度不断加大，东部电厂、LNG天然气发电厂和一批电厂综合配套工程建设加快。国家成品油储备深圳油库项目取得积极进展，西气东输二线工程正式开工，城市天然气输配管网项目建设有序推进，管道天然气转换提前完成，油气及其他危险品仓储搬迁整治工作进展顺利，成品油、天然气供应情况良好。

　　环境保护力度加大。在全国率先出台生态文明建设行动纲领等系列政策文件，成为国家生态文明建设示范地区，荣获首届“中国十佳绿色城市”称号。循环经济和节能减排工作深入推进。“蓝天行动”成效显著，按国家标准测定的全年环境空气质量优良天数达到364天。污水、污泥和垃圾处理等治污保洁工程加快推进，饮用水源和主要河流水质持续改善。基本生态控制线保护继续加强，生态风景林建设任务全面完成。大力发展绿色建筑，建筑节能水平进一步提高。

　　（六）民生福利水平持续提升。全市居民人均可支配收入达到26729元，增长10%。促进失业人员就业3.4万人，登记失业率控制在2.3%。劳动关系总体和谐稳定，欠薪保障制度不断完善，劳动用工关系进一步规范，劳动合同签订率达到98.1%。最低工资和最低生活保障标准大幅提高。社会保障覆盖面继续扩大，劳务工医疗保险参保人数达610.2万，少儿医保和统筹医疗参保人数达51.7万，农民工养老、工伤和医疗保险参保数量均名列内地大中城市之首，初步实现全民医保。我市关爱农民工工作受到国务院表彰。企业退休人员基本养老待遇人均每月增加511元。全年为低收入家庭新提供2726套保障性住房。

　　全面推行义务教育双免，在国家标准基础上增加免费项目，扩大覆盖范围，受益学生达到77.2万人次，累计免费3.22亿元。继续推进义务教育均衡化发展，加快寄宿制高中建设，促进学前教育规范健康发展，积极调研解决“午托班”、临聘教师问题，特区外96所原村办小学校舍改扩建工程进展顺利。加快发展高等教育，南方科技大学动工建设，深圳大学等市属高校办学水平不断提高，大学城功能进一步发挥。民办教育和职业教育规范发展，终身教育体系进一步完善。国际学校建设取得突破性进展。深入实施卫生事业“一大一小”发展战略，市眼科医院新院、急救中心指挥调度系统等投入使用，市第三人民医院、疾病预防控制中心主体结构工程完工，滨海医院动工建设，宝荷医院、新安医院正式奠基。新建30家社区健康服务中心，社区健康服务网络基本实现全覆盖。公共文化服务体系进一步完善，博物馆新馆、少儿图书馆建成开馆，24小时街区自助图书馆系统投入使用。纪念改革开放30周年理论研究和文学工程硕果累累，沙头角鱼灯舞、平乐郭氏正骨医术列入国家级非物质文化遗产名录，“深圳读书月”、“关爱行动”、“市民文化大讲堂”、“科技大讲堂”、“公园文化节”等品牌文化活动蓬勃开展，蝉联全国文明城市称号，正式加入全球创意城市网络，被联合国教科文组织评为“设计之都”。大运会场馆建设顺利推进，筹备团队运作高效，宣传活动广泛开展，市场开发有所突破，大运会进入全面筹备阶段。出色完成北京奥运会和残奥会火炬传递活动，我市运动员在奥运会和残奥会上夺得一金两铜的历史最好成绩。

　　深入推进社会治安综合治理，社会治安形势总体向好。全面加大矛盾纠纷排查化解力度，不断完善“大调解”体系，信访维稳工作有序推进，市政府12345公开电话15秒内接通率达到97%，为群众解决了一大批实际问题。大力加强城市应急管理和灾害防治，完善应急平台，强化安保和反恐工作，妥善处置各类突发公共事件。深刻吸取“2·27”、“9·20”火灾事故教训，扎实开展安全隐患大排查、大整治等行动，全面加强安全生产管理制度建设，弥补了38个方面的制度缺失。安全生产各类事故数和死亡人数分别比上年下降24.9%和8.7%。食品安全“五大工程、四大体系”建设稳步推进，基本完成人大关于加强食品安全监督管理议案确定的各项目标任务，药品安全抽样合格率首次超过97%。妥善处理了三聚氰胺奶粉事件。率先建立起比较完善的人口计生公共服务体系，户籍人口和流动人口政策生育率分别达到98.9%和92.2%，六区均被评为全国计划生育优质服务先进区。第五届居委会选举圆满完成，直选率达92.8%。社会福利、慈善、老年人和残疾人事业继续发展。国防建设、民兵预备役和军民共建工作扎实推进，第六次荣获广东省“双拥模范城”称号，六区首次全部评为广东“双拥模范区”。档案、外事、侨务、港澳、台务、民族宗教、统计、气象、民防等各项事业都取得新进步。

　　一年来，我们努力推进政府自身建设，依法行政水平进一步提高。认真执行市人大及其常委会决议，坚持向人大常委会报告工作，及时向市政协常委会通报政务，自觉接受市人大的法律监督、工作监督和市政协的民主监督。办理市人大重点建议2件，人大代表建议617件；政协建议案3件，政协提案669件，代表和委员满意和基本满意率达到99.5%以上。法治政府建设取得重大突破，制订法治政府建设指标体系，与国务院法制办签订了加快法治政府建设合作协议，出台加快法治政府建设的2009年市政府1号文件，提交市人大常委会审议的地方法规和特区法规草案12项，颁布规章21项。行政执行力进一步提高，根据《政府工作报告》分解的159项主要工作经认真督查督办和强力协调，全部按计划完成。行政审批电子监察系统绩效测评保持全省第一，行政服务大厅业务量位居全国同类机构首位，业务按时办结率达100%。继续完善行政责任“白皮书”制度，积极推进行政问责，全年责任追究199人、13个单位。加快推进政务公开和电子政务建设，市政府门户网站在全国地市级（含副省级）城市政府网站绩效评估中名列第一。

　　各位代表，在过去一年异常困难的形势下，我们取得这些成绩，实属来之不易！这要归功于党中央、国务院和省委、省政府的坚强领导和亲切关怀，归功于历届市委市政府团结带领全市人民奋力拼搏打下的坚实基础，归功于全市人民特别是广大企业和员工毫无畏惧、迎难而上、同舟共济、共克时艰的精神和勇气。在此，我代表深圳市人民政府，向全市广大干部群众和外地来深建设者表示最崇高的敬意！向人大代表、政协委员，向各民主党派、社会团体和各界人士，向中央和各省区市驻深单位，驻深解放军、武警官兵，向所有关心支持深圳建设的港澳同胞、台湾同胞、海外侨胞和国际友人，表示最衷心的感谢！

　　我们也清醒地看到，我市经济社会发展还存在一些亟待解决的困难和问题：在新的形势下，科学平衡经济稳定增长和产业转型升级之间的关系更加困难，资源短缺和环境约束的压力进一步加大；经济问题加速向社会领域渗透，多年来积累的体制机制性矛盾和深层次问题更加突出，社会维稳形势不容乐观；建立安全生产的长效管理机制任重道远，各种事故隐患尚未根本消除，重大安全事故时有发生，安全生产形势依然严峻；农村城市化遗留问题尚未根本解决，特区外基础设施历史欠账较多，特区内外一体化发展仍然需要付出巨大努力；政府公共服务水平有待进一步提高，一些群众关心的热点难点问题还没有得到有效解决，等等。对这些问题，我们将以高度的责任感和使命感，强力推进解决，绝不辜负人民的期望！

　　二、2009年主要工作

　　2009年，国际国内形势将更为复杂，国际金融危机正在加速演变成为一次全球性的经济危机，国内经济运行困难增加。深圳作为经济国际化程度高、出口依存度大的沿海开放城市，受影响和冲击比其他地区更大，今年是我市进入新世纪以来经济形势最为困难的一年。危机既是挑战和压力，更是落实科学发展观、加快转变发展模式的机遇和动力；危机可能会带来很多改变，但我国经济发展的基本面和长期趋势没有改变，深圳经济特区的精神和根基没有改变，市委市政府深化改革开放推动科学发展的目标和决心没有改变。特别是《珠江三角洲地区改革发展规划纲要》的出台，第一次将珠三角的改革发展整体纳入国家战略规划层面，进一步赋予了深圳新使命、新定位，为深圳的改革开放和现代化建设注入了强大动力。我们遇到的困难和问题是前进中的问题、转型中的阵痛。只要我们统一思想、振奋精神、坚定信心，将形势考虑得更严峻一些，把困难估计得更充分一些，把措施准备得更周密一些，以积极进取的精神迎接挑战，就一定能够攻坚克难、战胜危机，确保深圳的科学发展不因危机而停步，改革开放不因危机而放慢，产业转型不因危机而中断，社会稳定不因危机而动摇，就一定能够超越自我，勇度难关，在新一轮发展中抢占先机，在科学发展的道路上实现新的跨越！

　　2009年政府工作的总体思路是：高举中国特色社会主义伟大旗帜，以邓小平理论和“三个代表”重要思想为指导，以科学发展观统领经济社会发展全局，按照市委四届十一次全会的总体部署，深入实施《珠江三角洲地区改革发展规划纲要》，加快推进综合配套改革，在珠三角新一轮发展中发挥示范带动作用；深入实施“一保四抓”，扩大内需外需，加强自主创新，推进产业升级，确保经济平稳较快发展；深入实施法治政府建设指标体系，以法治政府建设带动法治城市建设，促进社会和谐稳定；深入推进十项重点工作的突破和十件民生实事的落实，努力提升民生福利水平，全面完成本届政府承诺的各项工作，为强化深圳国家经济中心城市和国家创新型城市地位，加快建设中国特色社会主义示范市和现代化国际化城市作出新贡献。

　　2009年经济社会发展的主要预期目标和约束指标是：本市生产总值增长10%左右；人均GDP增长9%左右；城镇居民登记失业率控制在3%以内；居民消费价格指数控制在103%左右；万元GDP能耗、水耗分别下降2.75%和4%；二氧化硫、化学需氧量排放总量分别下降2%和6%。

　　围绕经济社会发展目标任务，今年将着重抓好八个方面的工作：

　　（一）千方百计扩大需求，确保经济平稳增长。以只争朝夕的精神，全面加快有利于提升城市竞争力、辐射力、影响力的重大基础设施项目，有利于城市经济可持续发展的重大产业项目，有利于解决市民关心的热点难点问题的重大民生项目的规划建设。全年政府投资计划安排项目842个，年度投资总规模596.6亿元，以高强度的政府投资引导带动全社会固定资产投资超常规增长。积极推进集成电路芯片、化合物半导体、TFT-LCD面板、流感疫苗、清洁能源汽车等在建产业项目加快建设；全面加快地铁1—5号线，盐田港区扩建、大铲湾港二期、机场扩建、南坪快速二期、丹平路一期、外环高速、沿江高速等交通设施建设；大力推进新安医院等市属医院、大运中心、中心公园、当代艺术馆、城市规划馆、档案中心等民生项目建设。紧紧抓住国家和省扩大投资的契机，加快推进广深港客运专线、厦深铁路、穗莞深城际线、岭澳核电、平安国际金融中心等项目建设，抓好深圳沿海防护林工程等九个中央国债项目的实施，争取更多的国家性、区域性重大投资项目落户深圳。加快投融资体制改革，创新项目建设管理模式。改革政府投资项目审批制度，实施并联审批，缩短项目投资管理链条。强化政府投资项目责任制和重大项目分级协调机制，严格落实项目动态管理制度，加快各项前期工作，切实推动项目建设进度。认真做好督促检查工作，强化对投资项目全方位、全过程的监督检查和跟踪审计，确保所有工程都经得起历史的检验和人民的评判。

　　积极扩大居民消费。研究制定鼓励消费的措施，努力消除一切不利于消费的政策壁垒，提升消费对深圳经济的贡献率。深化收入分配制度改革，建立完善全社会工资性收入正常增长机制，提高劳动报酬在初次分配中的比重。促进居民财产性收入的增加，提高居民收入预期。完善医疗、教育、养老等基本保障体系，增强居民消费信心。引导房地产市场规范、健康发展，促进住房、汽车等大宗商品消费。在政府采购、政府投资项目和工程建设中，加大对本地自主品牌企业的支持力度。以国家推行“国民休闲旅游计划”为契机，加快发展旅游经济，鼓励市民增加旅游、餐饮、娱乐、休闲等消费支出，努力扩大节假日消费规模。加快华强北、罗湖金三角等老商业区改造升级，积极培育市中心区、南山商业文化中心、特区外中心城区等新兴商业旺区。加快发展连锁商业、信贷消费等新型业态，大力发展电子商务，培育一批在全国有影响力的电子商务企业。进一步加强市场监管，坚决打击假冒伪劣等违法行为，加快建立统一的社会信用体系和信息公共平台，建立失信惩戒和守信激励机制，形成开放有序的市场经营和消费环境。

　　全力开拓国内外市场。积极落实国家出口退税和加工贸易台账实转空制度，研究制定进一步鼓励外贸出口的政策措施。加快转变外贸发展方式，优化产品出口结构，大力发展服务贸易，积极开拓国际市场特别是新兴市场，努力保持出口形势的稳定。高度重视经济危机中贸易摩擦加剧的趋势，完善应对工作机制，加强对应诉企业的指导和支持，发挥好反倾销和产业损害预警系统的作用，帮助企业用好用足我国对外谈判的成果。大力实施内贸战略，设立加工贸易扩大内销专项扶持资金，发挥好驻外办事处、深商服务中心和行业协会作用，积极搭建各种平台，鼓励企业建立健全国内市场营销渠道和网络，引导企业开拓国内市场。

　　（二）坚定不移地深化改革，着力扩大对外开放。高度重视、认真把握《珠江三角洲地区改革发展规划纲要》给深圳带来的重大发展机遇，加强学习、统一认识，逐条分解《纲要》内容，研究提出落实举措，抓紧制定配套政策，深入推进《纲要》落实。充分发挥深圳经济中心城市的辐射力和带动力，全面加强与珠三角其它城市的合作，推动建立深莞惠区域协调发展联动机制，努力形成优势互补、良性互动的区域经济发展新格局，以服务全省、服务全国创造深圳发展的新空间。

　　积极稳妥地推进行政体制改革。着力转变政府职能，按照职能有机统一的原则，优化政务流程，整合政府机构，完善大部门管理体制，理顺运行机制。积极探索精简行政层级改革试点，缩短管理链条，提高行政效率。进一步调整市、区、街道和职能部门之间的事权划分，减少事权的重叠和交叉。积极推进全市行政审批服务体系和“在线审批系统”建设，精简项目，简化程序，再造流程，全面提高审批效率。积极推进公务员分类改革试点和聘任制公务员制度试点，深入推进公安专业化改革，支持配合法官、检察官职业化改革方案的论证和研究。

　　继续深化经济体制改革。进一步优化调整国有经济战略布局，完善国有产权制度，创新国有资产运营和管理模式。加强国有独资企业董事会制度建设，健全法人治理结构。深化国有文化资产管理体制改革，按照“效益集团”的要求，完善考核体系，促进国有文化集团做大做强。以实现基本公共服务均等化为目标，进一步深化财政体制改革，加快建立公共财政体系，加大特区外社会民生投入，促进特区内外协调发展。继续推进投融资体制改革，建立完善社会投资项目核准备案制度，鼓励社会资本进入金融服务、公用事业和基础设施等领域，形成有序竞争的格局。积极探索环境权益交易，加快建立生态环境补偿机制。

　　积极推进公共服务和社会体制改革。加快公共服务市场化、社会化，大力发展社会组织，支持社会组织拓宽发展空间，加快建立和完善政府向社会组织购买服务的制度。继续深化事业单位管理体制和运行机制改革，创新公用事业监管模式，推动公用事业监管体制改革。创新人口管理方式和手段，全面推行居住证制度，推动户籍制度和流动人口管理改革。加快推进医疗卫生体制改革，实行政事分开、管办分开、医药分开、营利性和非营利性分开。开展公立医疗机构、医疗保险和药品流通体制改革试点，有效增加基本医疗服务供给，逐步形成由政府提供公益性基本医疗服务、市场提供个性化和高端医疗服务的格局。继续深化教育体制改革，扩大各级各类教育的开放和准入程度，创新高校内部管理体制和运行体制，争取成为“全国教育综合改革试验区”。

　　积极扩大对外开放。继续实施“走出去”战略，支持企业积极稳妥地开展收购兼并，完善全球布局，实现低成本扩张。进一步提升对外交流合作水平，积极推进中国-越南（深圳-海防）经贸合作区建设，努力打造全国境外合作区的样板项目。继续探索深港、深澳合作新机制、新模式，加快推进落马洲河套地区开发和莲塘/香园围口岸、东部跨境高速公路等重大跨境基础设施建设，推动与香港共同编制区域合作规划，促进深港融合发展，建设与香港共同发展的国际性城市。积极推进与汕尾共建省示范性产业转移工业园，认真做好甘肃“三县一区”的对口支援恢复重建工作，加强对贵州、西藏、重庆、广东河源等地的对口扶持。

　　（三）大力推进自主创新，加快构建现代产业体系。全面落实建设国家创新型城市的总体规划和各项政策，抓紧出台具体实施细则、配套措施和操作流程，加强政策解读和督查督办，切实推动政策落地、资金落地、项目落地。加大政府研发投入和重大专项投入，确保未来三年内科技研发资金每年增加3亿元，今年针对企业的科技计划资金上半年全部发放到位。加大国家重点实验室、国家工程实验室和公共技术平台建设力度，鼓励和支持大学、科研机构和企事业单位积极申报国家和省科技计划，积极参加国家重大科技专项，吸引科技重大基础设施落户深圳，深化与国家部委的创新联动机制，在更大范围和更深层次聚集国家创新和科技资源。着力培育和吸引创新的技术源、项目源和企业源，设立产业技术攻关计划，力争在一些制约重点产业发展的关键技术和共性技术上取得新突破，切实增强源头创新能力。抓紧开展高新技术企业认定工作，严格落实国家有关企业研发费用税前扣除优惠政策，加大科技创新企业上市力度，加快建设创新型产业用房，为创新企业提供更大的发展空间。大力实施知识产权战略、标准战略和品牌战略，深入推进国家知识产权示范城市建设，切实增强深圳企业和产业的标准话语权。培育一批知名品牌，规范引导品牌效应低的初级创新产品生产企业转型升级，提升品牌，自主研发。强化深圳“改革之城”、“创业之城”的形象，优化创新创业环境，增强城市对人才的吸引力。完善人才政策，推进人才载体建设，提高人才待遇，降低生活成本，加大高层次人才队伍建设和海外人才招聘力度，着力打造完整的创新人才链。积极推进“深港创新圈”建设，促进两地创新资源整合，加快形成更高水平的跨境区域创新体系及科技创新产业聚集区。努力推进科技成果转化，办好第十一届高交会。

　　加快四大支柱产业发展。今年财政安排产业发展资金90.8亿元，同比增长60%，着力打造“高端化、集群化、总部型、创新型”现代产业体系。加快建设综合性国家高技术产业基地，大力发展以自主创新为特征的高新技术产业，突出电子信息产业的龙头地位，最大限度地集聚资源，完善布局，优化环境，努力建设世界级电子信息产业城市。加快落实LED产业发展规划和产品示范工程，推动LED产业做强做大。积极发展下一代互联网、IC设计、机器人、航空航天、新能源、新材料、生物医药、邮轮游艇等新兴产业，加快发展汽车及汽车电子、机电一体化等装备制造业和精细化工业，培育新的产业增长点。加快金融中心区和金融产业基地建设，吸引更多海外金融机构及后台服务机构落户深圳。积极完善多层次资本市场，培育创业板上市资源，优化基金发展环境，促进风险投资和私募股权投资基金的集聚。加大金融创新力度，落实和推进与港澳货物贸易人民币结算试点工作，培育企业债券市场，加快推进保险创新发展，完善中小企业融资服务体系，发展小额贷款公司和创新型金融机构，努力建设全国金融改革创新综合实验区。积极发展现代物流业，打造全国最优物流服务城市。大力拓展内贸业务和国际中转业务。加强深港机场合作，不断拓展航空客货运市场。加快前海湾保税港区建设发展，促进盐田港区向综合保税区转型升级。加快物流园区发展，推进物流业向供应链转型升级，努力把深圳建设成为亚太地区重要的多式联运中心和供应链管理中心，强化全球物流枢纽城市地位。深入实施文化产业发展规划纲要，推动设立文化产业投资基金，打造一批文化领军企业。充分挖掘“设计之都”品牌资源，积极吸引创意资本和人才，开展全民创意行动，加快规划建设国家级创意产业园区，推动“两城一都一基地”建设。提高第五届文博会的国际化、专业化水平，积极申办2010年世界创意大会。大力实施科技促农兴农，发展现代都市农业。培育发展适合深圳特点的海洋经济和海洋产业，努力打造海洋强市。扎实推进国家循环经济示范城市建设，大力发展循环经济，建立健全资源节约标准体系，加快构建节能环保型产业体系。

　　抓紧优化产业结构和产业布局。积极推动高新技术产业与传统产业的融合渗透，以高新技术改造提升优势传统产业。加快构建加工贸易转型升级服务平台，探索完善加工贸易企业“不停产原地转型”模式。大力推动电镀、线路板等高污染行业转型升级。准确把握产业转型升级中的平衡，坚持先长后消、长大于消，积极稳妥地推动产业有序转移。以国际一流标准，加快推进前后海地区规划建设，全力推动高新园区、光明新区和大工业区的发展建设，并以此为带动，积极推进精细化工生态产业园、国家生物产业基地、航天科技产业基地以及九大传统产业集聚基地建设，全面提升产业的集群化水平。

　　（四）大力发展各项社会事业，努力提高民生福利水平。越是经济困难，越要高度重视民生，切实改善民生福利。加快推进义务教育均衡发展，合理配置义务教育办学资源，进一步做好义务教育“双免”工作。继续扩大优质教育资源，全面落实素质教育，不断提升办学质量。加快筹建南方科技大学，进一步提升市属高校教学科研水平，完善大学城办学机制，鼓励香港高等教育机构来深办学。开展学前教育综合改革试验，大力发展职业教育和民办教育，积极推进终身教育。加强国际教育交流与合作，加快国际学校和港人子弟学校建设。

　　努力培育城市文化，增强城市凝聚力，提升市民的家园意识，强化市民的责任感、光荣感、幸福感和归属感，共同维护城市荣誉。深化文化体制改革，建立覆盖全社会的公共文化服务体系，全面提升文化软实力。完善公益性文化事业扶持政策，鼓励社会力量参与公益性文化建设，加强公益广告宣传力度。大力实施文艺精品战略，围绕建国60周年等重大题材，推出一系列反映时代精神、具有深圳特色的文艺精品。加强文物保护，加大对客家围屋、南头古城等文物的保护开发。推进全市有线电视网络重组整合，加快实施特区外有线电视数字化改造。加强文化市场管理，加大“扫黄打非”力度。进一步巩固文明城市创建成果，开展文化、健康、公德、环保、服务和爱心素质提升六大人文工程，不断提高市民素质。全力做好大运会各项筹备工作，积极推进大运会“城市行动计划”的实施，广泛开展群众性体育活动，为迎接大运会营造良好的社会氛围。

　　继续实施卫生事业“一大一小”发展战略，探索设立区域卫生医疗中心，促进市级医院合理布局，让全市人民都能就近享受到高水平医疗服务。深化社区卫生服务改革，加快社康中心原有物业回购进程，增强社区健康服务的功能。充分发挥中医在预防保健中的独特优势，推动中医药事业发展。促进基本公共卫生服务均等化，完善卫生应急机制，提高突发公共卫生事件应对水平。

　　大力实施积极就业政策，完善就业促进机制，全力打造充分就业城市。推进以创业促进就业，加强劳动者技能素质培训，做好就业困难、“零就业家庭”人员就业援助工作。加强就业管理基础工作，全面推进就业登记制度。积极构建和谐劳动关系，全面落实劳动合同制度，依法保障劳资双方合法权益。不断完善社会保障体系，提高社会保障水平，扩大参保人数，做实个人账户，力争在全国率先实现“全民社保”。逐步健全以扶老、助残、救孤、济困为重点，以家庭为基础、社区为依托、机构为补充的社会福利服务体系，积极稳妥推进社会福利向普惠型发展。以“人人享有基本生活保障”为目标，加快建立健全以最低生活保障制度为核心的综合性社会救助体系。设立第二保障线，对低保边缘群体实施分层分类专项救助。特别注重解决好困难群体和低收入群体的生活问题，建立困难群体生活补贴与物价水平联动机制，确保他们的生活水平不因物价上涨、经济下滑而明显降低。加快推进住房公积金制度改革，加快经济适用房建设，进一步健全多层次、多渠道的住房保障体系，逐步扩大住房保障覆盖面。

　　进一步完善社区管理体制，继续强化基层基础建设，不断提升基层自治水平。加强流动人口计划生育服务管理，巩固计生工作成果。完善优抚安置保障体系，巩固和加强军政军民团结。继续加强统计、外事、侨务、港澳、台务、民族宗教、防灾减灾、气象、民防、档案等工作。

　　（五）高度重视社会稳定，积极建设平安深圳。密切关注和防范经济形势变化可能引发的不安定因素，建立健全经济社会安全预测预警系统，提早做好风险监控及应对预案，确保经济安全运行。进一步加强金融监管，及时发布金融风险提示和预警，建立突发事件应急机制，严厉打击金融投机和恶意炒作等违法违规行为，有效防范和规避金融风险。严厉打击恶意欠薪和欠薪逃匿行为，进一步完善欠薪保障制度，促进劳资协商共度难关。妥善处理城市发展历史遗留问题，避免引发群体性事件。进一步健全和落实信访维稳工作责任制，加快构建“大排查”、“大调解”、“大信访”工作格局，加强对农民工法律援助工作，深入开展领导干部“大接访”和“直通车”、“民心桥”等活动，及时化解各类社会矛盾。加强法制宣传教育，坚决处置暴力维权和违法维权行为，引导群众依法、理性表达诉求，维护法律尊严。加强应急管理工作，构建群防群治突发事件管理机制，提高各级政府应对突发公共事件的能力。

　　加大社会治安综合治理力度。深入推进禁摩、视频监控等措施，不断提升社会治安综合治理水平。以城中村和老屋村为重点，对治安隐患进行逐个整治，实现治安总体形势继续好转。进一步强化打私、禁毒工作，保持对“黑恶势力”、严重暴力犯罪的严打高压态势，大力打击“两抢两盗”等影响恶劣的犯罪行为，提升群众安全感。进一步加强反恐工作，抓紧建立“情报、打击、防范、应急”四位一体的反恐实战机制，着力提高反恐实战能力。

　　切实加强安全生产和食品药品安全工作。认真吸取“2·27”、“9·20”火灾事故教训，扎实开展“安全生产年”活动，坚持做到安全生产警钟长鸣、常抓不懈、永不言胜。不断完善安全生产体制机制，加强对重点行业和领域的安全监管，做好安全隐患排查整治工作，防范重特大事故发生。创新食品药品安全监管体制，完善监管体系，加强安全风险评估，加大安全治理力度，不断提高老百姓的饮食、用药安全水平。建立安全宣教长效机制，不断增强广大市民的安全意识。

　　（六）努力提高城市品位内涵，不断完善城市功能。以筹办大运会为契机，以世界先进城市为标杆，着力提升城市规划建设管理水平。科学谋划城市发展布局，加大规划编制报批力度，力争城市总体规划和土地利用总体规划年内通过国家审批。加快制定总体规划实施纲要，完善实施机制，加大实施力度，抓紧将规划转化为具体的建设项目。以光明新城等四大新城建设为核心，以提高特区外基础设施水平为重点，全力促进特区内外一体化发展。突出城市风格特色要求，继续推进城市重点地段、重点区域城市设计和标志性建筑设计，高度关注小尺度空间和城市细部的设计，对城市街景、建筑物、公共空间及门户地区实行艺术化设计和人性化改造，集中打造一批建筑精品和城市亮点。全面完成深圳湾15公里滨海休闲带堤岸填筑工程，加快大鹏湾20公里海滨栈道建设，进一步增强现代化滨海城市特色。

　　着力强化和创新土地管理。继续完善以规划实施为导向的建设用地管理模式和土地计划调控机制，建立健全土地协同管理责任机制和土地执法长效机制。严格落实耕地保护责任，加快基本农田保护区的土地开发整理，确保我市基本农田在2010年前通过国家验收。逐步实行土地弹性年期制度，借鉴香港勾地模式，完善产业用地出让方式。进一步完善征地拆迁补偿的评估机制和补偿标准。建立健全建设用地集约利用评价考核指标体系，结合城市更新改造，加快存量土地的二次开发，继续加大闲置土地和土地历史遗留问题的处理力度，加大地下空间开发研究，持续提高土地资源的节约集约利用水平。

　　扎实推进生态文明建设。完善和强化环境与发展综合决策机制，积极推进规划环评、政策环评等战略环评。严格实施环保实绩考核，切实强化各区、各部门环保责任。积极实施大运会生态环境保障主体行动计划，认真做好国家环保模范城第三次复查迎检准备工作。在环保标准不降、要求不松的前提下加大管理和服务力度，注重技术引导和资金扶持，帮助企业清洁生产、合规经营。扎实推进污染减排工作，严格控制重点污染源，确保国控、省控和市控污染源达标排放。深入开展排水管网清源行动和水源保护行动，加快推进水环境综合治理，确保2010年前水环境质量有根本改善。持续推进“蓝天行动”，争取全市机动车和车用燃油全面实施国Ⅳ标准，积极推动电动汽车产业发展。继续推进南头半岛、清水河等重点区域环境综合整治。加强基本生态控制线监测管理，推动“四带六廊”生态安全网络体系建设。加强区域环境保护合作，协调解决跨境、跨界等区域污染问题。大力发展绿色建筑，积极落实建筑节能改造计划。持续推进绿色创建活动，积极引导绿色消费，大力提升全社会生态文明意识。

　　努力提高城市管理水平。积极借鉴国际先进城市管理经验，着力提高城市管理的精细化、规范化和信息化水平。继续推进城市环境综合整治，实现全市城中村和社区环境综合整治基本达标，特区内各区和特区外各街道各完成一条街的街景整治工作。加快大鹏半岛国家地质公园和儿童乐园、安托山等一批城市公园建设，新建20个社区公园。进一步加强环境卫生管理，规范垃圾转运站的运营管理。以道路修缮、路灯整治工作为重点，继续推进市政设施综合整治工作。深化公交特许经营改革，进一步优化公交经营机制。深入实施三层次公交线网规划，新开通一批快、干、支线路。稳步推进出租车运价调整及运力投放。加大非法营运、套牌车打击力度，加强泥头车管理，建立长效管理机制。

　　加大资源能源储备力度。积极利用全球资源价格下跌的机会，加大对水电油煤气等资源能源的储备。进一步增强水资源战略保障能力，继续推进公明、铁岗、清林径、下矶角等水库建设和清淤扩容。强力推进电网建设项目，今明两年力争完成250亿元电网建设投资，从根本上解决深圳用电瓶颈问题。加快深圳滨海电厂、抽水蓄能电站等项目建设，积极推进垃圾焚烧发电、风力发电、太阳能光伏系统等新兴能源项目的研究论证和规划建设。加快实施石油替代战略，拓展天然气资源供应渠道，积极推进西气东输二线、深圳LNG、城市天然气高压输配管网、国家成品油储备深圳油库等项目建设。加强粮食等重要物资的国家储备和商业储备。

　　（七）着力强化公共服务，积极建设法治政府。认真落实与国务院法制办签订的加快建设法治政府合作协议，抓紧实施法治政府建设指标体系，全力推进依法行政，力争用三年时间初步实现国务院规定的法治政府建设目标，树立法治政府、责任政府形象和权威，带动全社会法治观念的增强和法治水平的提高。创新政府立法工作机制，优化整合政府立法资源，逐步实行法规规章集中草拟，切实提高立法质量。深入推进行政综合执法，理顺街道综合执法机制，加强部门协作和执法保障，规范执法程序，提高执法水平。建立健全决策咨询机制，完善重大决策的社会参与、专家咨询、听证和公示制度，推进决策民主化、科学化。认真接受市人大及其常委会的法律监督和工作监督，坚持定期向市人大常委会报告重要工作。认真接受市政协的民主监督，加强与市政协的工作沟通和协商，主动听取各民主党派、人民团体和社会各界的意见和建议。切实执行市人大及其常委会通过的各项决议，认真办理人大建议和政协提案、建议案，提高办理工作的质量和实际效果。

　　不断优化政府服务。今年政府工作的首要任务是保增长，关键在于抓落实。要按照市委市政府的统一部署，确保今年各项指标任务的顺利完成。深入开展“服务年”活动，进一步强化政府服务理念，努力转变机关工作作风，下决心解决文山会海问题，力争做到全年会议、文件、评比表彰活动“三减少”，腾出更多的时间和精力投入到保增长、保民生、保稳定。创新政府服务手段，建立健全政府联系企业、服务企业的长效机制。进一步提高行政服务大厅服务水平和整合能力，打造一流的综合行政服务机构。积极推行政务公开，加强电子政务建设，发挥市政府12345公开电话的沟通平台作用，努力打造阳光政府。完善政府绩效评估指标体系，逐步扩大绩效评估范围，积极开展绩效审计。

　　扎实推进廉政建设工作。认真贯彻落实胡锦涛总书记在第十七届中纪委三次全会上的讲话精神，树立和坚持正确的事业观、工作观、政绩观。毫不动摇地加强廉政建设和反腐败斗争，坚持标本兼治、综合治理、惩防并举、注重预防的方针，以廉政建设和反腐败斗争的新成效取信于民。加强教育和监督，加大查办案件和行政问责力度，着力解决重点领域的腐败问题，严肃查处滥用职权、贪污贿赂、腐化堕落、失职渎职和重大责任事故背后的案件。深入开展纠风专项治理，建立纠风工作长效机制。全面清理各种行政事业性收费和服务性收费，取消一切不合理收费，降低过高收费。全市政府部门要发扬艰苦奋斗、勤俭办事的精神，带头过紧日子，严格财政预算编制执行，严控出国出访考察，严控会议、接待、差旅等公务支出，实行一般性行政经费零增长。

　　（八）实现十大工作突破，办好十件民生实事

　　今年是本届政府最后一个工作年度，全体政府工作人员必须增强责任感、紧迫感，切实转变工作作风，求真务实，真抓实干，不漂浮、不作秀、不忽悠，脚踏实地、扎扎实实地推进各项工作，完成好本届政府的使命和各项承诺，不留败笔、不留遗憾、不留骂名，向全市人民交上一份满意的答卷。在广泛调研的基础上，我们自加压力，确定了10个重点领域或关键环节，通过科学部署和狠抓落实，力争取得实质性突破，解决一批长期悬而未决的问题，以此带动全局的发展。

　　一是国家创新型城市建设要有新突破。加快组建电子信息产品标准化等一批国家重点工程实验室和国家高技术产业创新中心，新建国家华南超级计算中心，筹备产业创新研究院，加快建设深圳科技中心。

　　二是招商引资工作要有新突破。设立投资推广署，整合全市招商引资资源。抓住国际经济调整和服务业转移的机遇，全面落实重大产业项目招商引资计划和招商引资责任制，瞄准世界500强和国内大型企业集团，以产业链高端环节、缺失环节和新兴战略性产业为突破口，以资本密集型、技术密集型、基地型、旗舰型项目为重点，引进一批符合现代产业体系要求的重大产业项目，进一步夯实产业发展基础。

　　三是法定图则覆盖面要有新突破。力争年内取得突破性进展，两年内实现规划建设用地法定图则全覆盖。妥善解决基本生态控制线内的历史遗留问题。

　　四是城市更新改造要有新突破。出台城市更新政策和总体规划，加快推进旧工业区升级改造试点工作，力争第一批项目全面开工，第二批项目加快推进；加快岗厦、大冲等城中村改造步伐；启动华强北等8至10个城市更新试点项目；加快鹿丹村等旧住宅区改造。

　　五是农村城市化历史遗留违法建筑和土地历史遗留问题处理要有新突破。抓紧制定出台处理农村城市化历史遗留违法建筑的政策措施，切实将违法建筑纳入法制化管理，加快推进各类土地历史遗留问题处理。

　　六是污水处理设施建设要有新突破。加快污水处理厂、污水管网和污泥处理等58项重点治污项目的建设，年内新建和改扩建污水处理厂14座，建成投入使用 5座，建成污水干管300公里，增加污水二级处理能力100万吨/日以上。建成南山污泥处理项目，开工建设老虎坑、上洋污泥处理中心。

　　七是轨道交通建设要有新突破。全年完成轨道交通建设投资190亿元，基本完成5条地铁线路土建主体工程，全面展开6个综合枢纽建设，推动完成广深港客运专线深圳北段土建工程，实现厦深铁路深圳段全面开工。

　　八是安全生产体制机制建设要有新突破。着力完善安全生产管理长效机制，加强安全生产基础建设，开发建设覆盖全市企业的安全监管信息化系统，完善安全生产网格化管理。加强安全宣传教育，年内投入使用现代安全实景模拟教育基地。

　　九是高等教育改革发展要有新突破。南方科技大学要力争通过教育部组织的考察评议；积极争取国家支持深圳大学探索有中国特色的现代大学制度；创新完善大学城办学管理的体制机制；推动香港高校在深独立办学取得实质性进展。

　　十是社区股份合作公司改革要有新突破。一区一试点，积极稳妥地启动社区股份合作公司改革，抓紧修订完善有关法规规章，进一步规范股份合作公司运作，逐步剥离社会职能，促进股份合作公司健康发展。

　　今年，要着眼于提高民生福利水平和解决群众关心的热点、难点问题，全力做好十件民生实事，让市民得到真正的实惠。

　　一是城镇登记失业率控制在3%以内，平均失业周期控制在6个月以下，当年新增失业人员、新成长劳动力和就业难人员就业率均达到80%以上。开发使用公益职业介绍网站，举办300场农民工免费职业介绍招聘会。

　　二是加快推进高中建设，增加5000个优质普高学位，全面完成特区外96所原村办小学改造，规范有序解决中小学生午餐午休问题，设立学前教育发展专项经费，积极稳妥推进公办中小学临聘教师问题的解决。

　　三是基本完成市第三人民医院、疾病预防控制中心工程建设，滨海医院、人民医院外科的主体结构实现封顶。每区规划建设1家以上市级医院。实行医疗机构药品零差价销售，降低药品价格。

　　四是建立完备的医疗保险体系，实施非从业居民社会医疗保险，确保全市常住人口医疗保险参保率达到100%。

　　五是落实新增保障性住房建设用地30万平方米，规划建设2.57万套保障性住房，加快53个保障性住房项目建设进度，着力解决低收入家庭住房难问题。

　　六是实现农产品生产基地主要农产品供应达到深圳市场的60%以上，市场豆制品全部从合法渠道进货，蔬菜农药残留、生猪肉品“瘦肉精”污染和水产品药物残留检测年平均合格率分别保持在96%、98%和98%以上。

　　七是加快打通全市范围内的断头路，积极推进交通智能化工程，统一梳理全市交通标识，加快建设口岸公交枢纽工程，新投放一批公交运力，基本解除特区外公交线路承包经营关系，初步完成特区内外公交线路网络化布局，特区外公交站点500米覆盖率达到75%以上。

　　八是力争年内实现所有符合条件的户籍居民都可申请办理一年多次往返香港旅游签注，所有符合条件的持居住证的非户籍居民都可申请在深办理赴香港“个人游”签注。

　　九是全面启动社区、住宅区和城中村消防设施、市政管线等改造，年内完成40%以上。实现特区内住宅区物业管理全覆盖，特区外原农村社区物业管理覆盖率达到50%。

　　十是加大全民健身公共体育设施投入力度，每年在100家以上社区公园配置体育设施，力争尽早实现全覆盖。有步骤向社会开放学校体育场馆，大力开展有组织的群众体育竞赛和健身展演活动，确保全年不少于1600次，市民体质合格率高于全国平均水平。

　　各位代表！

　　今年是新中国成立60周年和深圳建市30周年，做好今年的工作，任务艰巨，使命光荣。面对困难，我们无所畏惧，面对未来，我们信心百倍！让我们高举中国特色社会主义伟大旗帜，以科学发展观统领全局，在市委的坚强领导下，团结带领全市人民，进一步解放思想、改革创新，振奋精神、顽强拼搏，推动经济社会又好又快发展，勇当落实科学发展观的排头兵，以优异的成绩向建国60周年和建市30周年献礼！
